# Supplementary material for: Plasma methionine metabolic profile is associated with longevity in mammals
Source: Commun Biol. 2021 Jun 11;4:725. doi: 10.1038/s42003-021-02254-3 (PMC8196171; doi:10.1038/s42003-021-02254-3)
Supplement: Supplementary file 2 — Supplementary Information [file 42003_2021_2254_MOESM2_ESM.pdf]

Supplementary Figure 1. Pearson correlation between longevity and the ratio of TCA cycle intermediates/amino acids (**A**) and amino acids and TCA cycle intermediates (**B**). Minimum signification level was set at $p<0.05$. All metabolites were log-transformed in order to accomplish the assumptions of normality. Data values are obtained from 4 (mice and cows), 5 (rats, rabbits, guinea pigs, sheep, dogs, pigs, cats and horses) or 6 (humans) specimens.

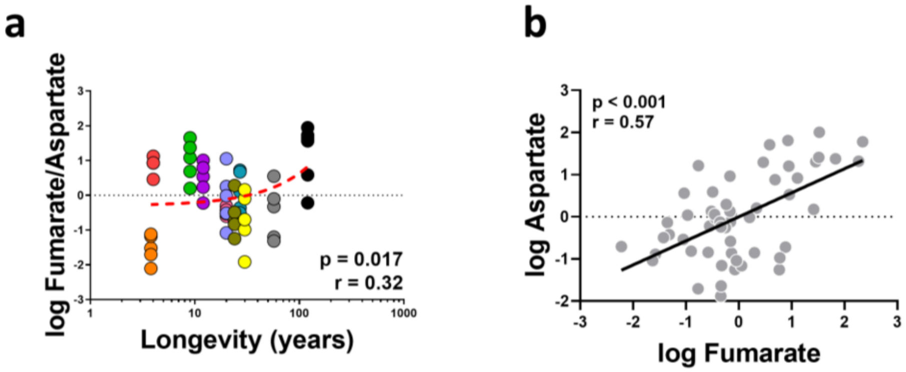

Supplementary Table 1. Analytical traits of the metabolites measured in plasma from mammalian species. Fragmentor, collision energy (CE) and cell acceleration voltage (CAV) are given as voltage; retention time (RT) in minutes; and product and precursor ion as m/z. Method 1 (see material and methods, Cabré et al. 2016); Method 2 (see material and methods, Liu et al. 2017). ND=Not detected metabolite.

Cpd Name	Prec Ion	Prod Ion	Frag (V)	CE (V)	Cell Acc (V)	Ret Time (min)	Ret Window	Polarity	Extraction	Method
**Amino acids**										
Alanine	90.06	44.2	40	8	7	0.376	2	Positive	Methanol	1
Arginine	175.1	70.2	60	20	7	0.32	2	Positive	Methanol	1
Arginine	175.1	60.2	60	15	7	0.32	2	Positive	Methanol	1
Asparagine	133	74.1	60	15	7	0.376	2	Positive	Methanol	1
Aspartate	134	43.2	60	15	7	0.362	2	Positive	Methanol	1
Aspartate	132	88.1	60	15	7	0.362	2	Negative	Methanol	1
Glutamate	146	102.1	60	15	7	0.363	2	Negative	Methanol	1
Glutamate	146	41	60	15	7	0.363	2	Negative	Methanol	1
Glycine	76.04	48	40	0	7	0.34	2	Positive	Methanol	1
Glycine	76.04	30	40	4	7	0.34	2	Positive	Methanol	1
Histidine	156	110.1	60	15	7	0.32	2	Positive	Methanol	1
Histidine	156	56.2	60	25	7	0.32	2	Positive	Methanol	1
Isoleucine	132.1	86	64	8	7	0.591	2	Positive	Methanol	1
Isoleucine	132.1	69	64	16	7	0.591	2	Positive	Methanol	1
Leucine	132.1	86	64	8	7	0.591	2	Positive	Methanol	1
Leucine	132.1	69	64	16	7	0.591	2	Positive	Methanol	1
Phenylalanine	164	147	100	15	7	0.841	2	Negative	Methanol	1
Phenylalanine	164	103.1	100	15	7	0.841	2	Negative	Methanol	1
Proline	116	70.2	60	15	7	0.392	2	Positive	Methanol	1
Serine	106.05	60	64	8	7	0.35	2	Positive	Methanol	1
Serine	106.05	42	64	24	7	0.35	2	Positive	Methanol	1
Serine	104.03	74	64	8	7	0.35	2	Negative	Methanol	1
Threonine	120	74.2	60	15	7	0.358	2	Positive	Methanol	1
Threonine	120	56.2	60	15	7	0.358	2	Positive	Methanol	1
Tryptophan	205	188.1	60	15	7	1.23	2	Positive	Methanol	1
Tryptophan	205	146.1	60	15	7	1.23	2	Positive	Methanol	1
Tyrosine	180.1	163.1	100	15	7	0.548	2	Negative	Methanol	1
Tyrosine	180.1	119.1	100	15	7	0.548	2	Negative	Methanol	1

Valine	118.08	72	64	8	7	0.43	2	Positive	Methanol	1
Valine	118.08	55	64	20	7	0.43	2	Positive	Methanol	1
**Methionine metabolism**										
Betaine	118.09	59.2	136	16	7	0.425	2	Positive	Methanol	1
Betaine	118.09	58.2	136	32	7	0.425	2	Positive	Methanol	1
Cysteine	122.02	76	64	12	7	6.312	4	Positive	DTT	2
Cysteine	122.02	59	64	24	7	6.312	4	Positive	DTT	2
Cystathionine	223.07	134	88	8	7	6.818	4	Positive	DTT	2
Cystathionine	223.07	88	88	28	7	6.818	4	Positive	DTT	2
Glutathione	308.09	179	88	8	7	0.5	2	Positive	DTT	1
Glutathione	308.09	76	88	24	7	0.5	2	Positive	DTT	1
Homocysteine	136.18	90.1	135	15	7	7.225	4	Positive	DTT	2
Homocysteine	136.18	56.2	135	15	7	7.225	4	Positive	DTT	2
Methionine	150.05	104	64	4	7	0.48	2	Positive	DTT	1
Pyridoxal	168.05	150	64	8	7	0.522	2	Positive	Methanol	1
Pyridoxal	168.05	94	64	24	7	0.522	2	Positive	Methanol	1
Pyrydoxal-5’-P (ND)	248.03	150	112	12	7	0.7	2	Positive	Methanol	1
Pyrydoxal-5’-P (ND)	248.03	67	112	32	7	0.7	2	Positive	Methanol	1
Pyridoxamine	169.09	152	64	8	7	0.366	2	Positive	Methanol	1
Pyridoxamine	169.09	134	64	20	7	0.366	2	Positive	Methanol	1
SAH	385.1	136	112	20	7	1.13	2	Positive	Methanol	1
SAH	385.1	88	112	48	7	1.13	2	Positive	Methanol	1
SAM	399.1	250	112	12	7	0.396	2	Positive	DTT	1
SAM	399.1	136	112	28	7	0.396	2	Positive	DTT	1
Spermidine (ND)	146.1	84	88	24	7	0.3	2	Positive	Methanol	1
Spermidine (ND)	146.1	72	88	12	7	0.3	2	Positive	Methanol	1
Taurine	126.02	108	88	8	7	0.38	2	Positive	DTT	1
Taurine	124	80	112	20	7	0.38	2	Negative	DTT	1
**TCA cycle metabolites**										
α-Ketoglutarate	145.01	101	64	4	7	0.435	2	Negative	Methanol	1
α-Ketoglutarate	145.01	57	64	20	7	0.435	2	Negative	Methanol	1
Citrate	191.01	111	88	8	7	0.637	2	Negative	Methanol	1
Citrate	191.01	87	88	16	7	0.637	2	Negative	Methanol	1
Fumarate	115.01	71	64	4	7	0.55	2	Negative	Methanol	1

Fumarate	115.01	27	64	4	7	0.55	2	Negative	Methanol	1
Isocitrate (ND)	191.01	111	88	8	7	0.393	2	Negative	Methanol	1
Isocitrate (ND)	191.01	87	88	16	7	0.393	2	Negative	Methanol	1
Malate	133.02	115	64	8	7	0.4	2	Negative	Methanol	1
Malate	133.02	71	64	12	7	0.4	2	Negative	Methanol	1
Pyruvate	87.01	43	64	4	7	0.413	2	Negative	Methanol	1
Succinate	117.02	73	64	8	7	0.57	2	Negative	Methanol	1
Succinate	117.02	55	64	20	7	0.57	2	Negative	Methanol	1
**Lipid intermediates**										
Carnitine	162.12	103.1	88	16	7	0.393	2	Positive	Methanol	1
Choline	104.11	60.2	112	16	7	0.39	2	Positive	Methanol	1
**ISTD**										
PheC13	167.09	120.1	70	8	7	0.87	2	Positive	Methanol/DTT	1/2
PheC13	167.09	77	70	44	7	0.87	2	Positive	Methanol/DTT	1/2
PheC13	167.09	103	70	28	7	0.87	2	Positive	Methanol/DTT	1/2
PheC13	167.09	51.1	70	60	7	0.87	2	Positive	Methanol/DTT	1/2

Supplementary Table 2. Plasma metabolite concentration in mammals. Concentration values are reported in MS Counts as mean ± SEM from 4 (mice and cows), 5 (rats, rabbits, guinea pigs, sheep, dogs, pigs, cats and horses) or 6 (humans) specimens.

	Mouse	Rat	Guinea pig	Rabbit	Sheep	Dog	Pig	Cat	Cow	Horse	Human
**Amino acids**											
**Alanine**	9622 ± 389	10872 ± 920	6132 ± 548	13653 ± 1114	7559 ± 704	13055 ± 975	16949 ± 835	16248 ± 2307	10007 ± 647	8534 ± 755	10395 ± 1112
**Arginine**	1547 ± 214	12298 ± 2446	7486 ± 1089	13396 ± 1465	19279 ± 1776	19709 ± 3070	28465 ± 3000	17738 ± 1448	14505 ± 1995	19389 ± 902	7862 ± 993
**Asparagine**	400 ± 33	482 ± 109	123 ± 20	456 ± 41	546 ± 73	637 ± 52	496 ± 59	442 ± 51	542 ± 73	102 ± 10	800 ± 83
**Aspartate**	300 ± 15	464 ± 52	444 ± 46	927 ± 48	499 ± 49	475 ± 23	818 ± 63	772 ± 75	521 ± 42	465 ± 32	486 ± 60
**Glutamate**	135± 14	423 ± 18	233 ± 18	173 ± 12	405 ± 31	206 ± 30	425 ± 32	458 ± 45	279 ± 34	285 ± 27	140 ± 9
**Glycine**	658 ± 59	845 ± 80	2176 ± 83	3092 ± 113	1336 ± 171	1415 ± 92	3226 ± 236	1437 ± 49	1550 ± 81	1692 ± 142	906 ± 43
**Histidine**	991 ± 144	1363 ± 274	3613 ± 235	5325 ± 164	2042 ± 191	3134 ± 198	4558 ± 221	3767 ± 100	3808 ± 507	2891 ± 274	3991 ± 306
**Isoleucine**	785631 ± 57318	1158881 ± 138350	3177542 ± 305357	4243780 ± 435646	3356794 ± 393084	2701301 ± 185822	6018470 ± 282885	3390951 ± 315707	3718411 ± 442245	2527144 ± 120129	3647776 ± 325288
**Leucine**	777564 ± 55554	1146902 ± 137694	3145356 ± 308314	4180307 ± 427257	3341866 ± 393766	2664909 ± 185321	5937102 ± 279439	3343632 ± 298778	3688931 ± 448074	2501138 ± 121446	3629458 ± 318561
**Phenylalanine**	1051 ± 96	1135 ± 175	2868 ± 157	2692 ± 147	2311 ± 155	2426 ± 195	5672 ± 180	3567 ± 219	3261 ± 275	2985 ± 87	3067 ± 154
**Proline**	20118 ± 1349	26539 ± 2882	58724 ± 4389	99020 ± 11006	35894 ± 3372	109229 ± 22987	221255 ± 27458	112626 ± 18397	93396 ± 13687	50925 ± 7538	127761 ± 16943
**Serine**	3256 ± 66	6054 ± 719	5016 ± 338	6643 ± 411	2481 ± 164	5940 ± 78	3954 ± 287	4601 ± 222	3689 ± 33	6413 ± 507	3030 ± 117
**Threonine**	3375 ± 228	6993 ± 479	3866 ± 457	9387 ± 372	3694 ± 580	9018 ± 1811	7905 ± 618	5715 ± 492	4121 ± 280	3024 ± 626	6077 ± 779
**Tryptophan**	56098 ± 5119	138813 ± 9949	428879 ± 34935	758001 ± 54933	365132 ± 41243	698597 ± 43142	833938 ± 94473	605752 ± 22566	504841 ± 47232	545571 ± 32168	628770 ± 41400
**Tyrosine**	218 ± 9	344 ± 53	319 ± 38	602 ± 61	390± 42	275 ± 16	886 ± 83	469 ± 23	454 ± 71	561 ± 22	515 ± 43
**Valine**	72467 ± 3425	87998 ± 5309	245806 ± 27796	469870 ± 49934	307657 ± 36262	226402 ± 6738	606992 ± 30600	202688 ± 18589	337455 ± 42436	255574 ± 11038	367707 ± 27965
**Methionine metabolism**											
**Betaine**	29479 ± 415	87359 ± 6785	103778 ± 19168	207317 ± 41281	182902 ± 10513	531171 ± 45601	586314 ± 41654	165751 ± 13196	464612 ± 39664	137181 ± 11397	76199 ± 8708
**Cystathionine**	2555 ± 16	1662 ± 155	816 ± 96	15928 ± 1305	4301 ± 708	10849 ± 1086	1766 ± 305	18531 ± 3913	5841 ± 581	451 ± 102	528 ± 186
**Cysteine**	224861 ± 6950	266951 ± 20243	296224 ± 19633	220210 ± 5763	73664 ± 16370	125619 ± 25690	229049 ± 22829	116156 ± 39559	37615 ± 8549	3649 0± 11370	352668 ± 17110
**GSH**	13867 ± 203	2044 ± 976	186 ± 69	630 ± 71	13 ± 2	594 ± 249	8 ± 2	1398 ± 637	21 ± 6	44 ± 9	118 ± 34
**Homocysteine**	816 ± 41	706 ± 66	387± 34	1476 ± 139	551 ± 153	2187 ± 724	1950 ± 279	919 ± 349	70 ± 19	ND	1102 ± 203
**Methionine**	9956 ± 1581	8094 ± 2542	2398 ± 390	9631 ± 996	1551 ± 251	830 ± 240	3062 ± 294	5613 ± 853	2027 ± 509	2161 ± 152	1513 ± 338
**Pyridoxal**	219 ± 24	203 ± 15	819 ± 46	1016 ± 68	959 ± 50	970 ± 16	1024 ± 30	965 ± 46	946 ± 39	937 ± 24	1020 ± 50
**Pyridoxamine**	689 ± 52	289 ± 41	2923 ± 317	287 ± 41	124 ± 6	333 ± 40	88 ± 10	198 ± 12	1178 ± 220	678 ± 49	9150 ± 254
**SAH**	77 ± 8	35 ± 7	257 ± 45	95 ± 14	226 ± 11	159 ± 23	355 ± 35	161 ± 12	69 ± 11	ND	135± 17

**SAM**	100 ± 30	53 ± 16	129 ± 42	112 ± 23	21 ± 4	21 ± 6	19 ± 5	51 ± 17	ND	ND	55 ± 12
**Taurine**	1142 ± 23	475 ± 98	257 ± 50	157 ± 27	186 ± 33	331 ± 40	378 ± 47	361 ± 22	285 ± 69	113 ± 22	209 ± 41
**TCA intermediates**											
**α-Ketoglutarate**	444 ± 29	53 ± 13	1408 ± 56	325 ± 68	1827 ± 200	42 ± 5	866 ± 238	112 ± 17	358 ± 72	145 ± 12	424 ± 60
**Citrate**	351 ± 22	99 ± 24	7014 ± 1539	21748 ± 4594	8248 ± 2351	4669 ± 907	2741 ± 101	6259 ± 697	7790 ± 2932	4819 ± 1134	1467 ± 268
**Fumarate**	163 ± 12	89 ± 9	199 ± 10	540 ± 52	181 ± 18	138 ± 15	323 ± 31	217 ± 34	158 ± 13	143 ± 16	306 ± 34
**Malate**	1124 ± 69	161 ± 19	1373± 127	3686 ± 605	1217 ± 246	519 ± 45	1714 ± 97	1067 ± 294	1126 ± 134	526 ± 65	369 ± 35
**Pyruvate**	448 ± 18	141± 20	246 ± 45	488 ± 67	266 ± 39	72 ± 6	247 ± 37	95 ± 9	220 ± 20	157 ± 24	454 ± 103
**Succinate**	6625 ± 620	1934 ± 323	13900 ± 2490	3551 ± 331	5771 ± 525	4453 ± 153	5643 ± 326	2441 ± 101	2172 ± 350	1774 ± 144	1249 ± 122
**Lipid intermediates**											
**Carnitine**	3534 ± 112	12662 ± 495	19196 ± 2994	12592 ± 3491	7061 ± 963	3197 ± 1012	2683 ± 828	5194 ± 474	4847 ± 1107	4946 ± 532	25626 ± 5850
**Choline**	191008 ± 10036	42815 ± 2620	130092 ± 6916	135483 ± 7654	49365 ± 11165	163835 ± 3927	113692 ± 5753	139621 ± 4642	124035 ± 11272	56938 ± 6904	47049 ± 1985
**TMAO**	1905 ± 296	1528 ± 103	8210 ± 767	19964 ± 3149	87395 ± 8160	16161 ± 788	18773 ± 1066	21143 ± 4081	12363 ± 2902	66200 ± 11286	22424 ± 4914

Supplementary Table 3. Plasma metabolite correlation with animal longevity. Pearson r values are reported. Pagels λ value measures phylogenetic signal for each individual metabolite. Phylogenetic generalized least squares (PGLS) regression between plasma metabolites and longevity corrects for phylogenetic relationships. Minimum signification level was set at $p<0.05$. All metabolites were log-transformed to accomplish the assumptions of normality. Data values are obtained from 4 (mice and cows), 5 (rats, rabbits, guinea pigs, sheep, dogs, pigs, cats and horses) or 6 (humans) specimens.

	**Pearson**		**PGLS**	
	***r* values**	**Sig.**	**λ**	**Sig.**
**Amino acids**				
Alanine	-0.02	0.861	0	0.922
Arginine	0.02	0.910	0	0.796
Asparagine	0.18	0.197	0	0.440
Aspartate	-0.07	0.633	1	0.871
Glutamate	-0.26	0.060	0.40	0.500
Glycine	-0.22	0.107	0	0.623
Histidine	0.31	0.022	0.93	0.391
Isoleucine	0.28	0.040	0	0.305
Leucine	0.28	0.038	0	0.300
Phenylalanine	0.33	0.015	0.37	0.302
Proline	0.37	0.006	0.72	0.258
Serine	-0.23	0.093	0	0.468
Threonine	-0.04	0.753	0	0.970
Tryptophan	0.37	0.006	0.79	0.334
Tyrosine	0.30	0.026	1	0.401
Valine	0.33	0.014	0	0.244
**Methionine metabolism**				
Betaine	-0.21	0.136	0	0.749
Cystathionine	-0.55	<0.001	1	0.140
Cysteine	0.04	0.768	0	0.981
GSH	-0.32	0.020	0	0.219
Homocysteine	0.10	0.487	1	0.902
Methionine	-0.38	0.004	0.19	0.155
Pyridoxal	0.39	0.003	0	0.195
Pyridoxamine	0.62	<0.001	0.42	0.045
SAH	0.18	0.193	0	0.625
SAM	-0.06	0.671	0	0.736
Taurine	-0.37	0.006	0.58	0.253
**TCA cycle metabolites**				
α-Ketoglutarate	0.05	0.732	0	0.917
Citrate	0.02	0.901	0	0.854
Fumarate	0.22	0.106	0	0.437
Malate	-0.34	0.012	0	0.350
Pyruvate	0.17	0.230	0	0.644
Succinate	-0.58	<0.001	0	0.045
**Lipid and protein intermediates**				
Carnitine	0.32	0.017	0	0.316
Choline	-0.44	0.001	1	0.481
