# Supplementary material for: Plasma methionine metabolic profile is associated with longevity in mammals
Source: Commun Biol. 2021 Jun 11;4:725. doi: 10.1038/s42003-021-02254-3 (PMC8196171; doi:10.1038/s42003-021-02254-3)
Supplement: Supplementary file 3 — Description of Supplementary Files [file 42003_2021_2254_MOESM3_ESM.pdf]

Description of Additional Supplementary Files

File name: Supplementary Data 1

Description: Individual values obtained from targeted analyses. Data is expressed in MS Counts.
