# Supplementary material for: Plasma methionine metabolic profile is associated with longevity in mammals
Source: Commun Biol. 2021 Jun 11;4:725. doi: 10.1038/s42003-021-02254-3 (PMC8196171; doi:10.1038/s42003-021-02254-3)
Supplement: Supplementary file 5 — Reporting Summary [file 42003_2021_2254_MOESM5_ESM.pdf]

nature research

Corresponding author(s): Prof. Pamplona, Reinald

Last updated by author(s): Aug 27, 2020

Reporting Summary

Nature Research wishes to improve the reproducibility of the work that we publish. This form provides structure for consistency and transparency in reporting. For further information on Nature Research policies, see our Editorial Policies and the Editorial Policy Checklist.

Statistics

For all statistical analyses, confirm that the following items are present in the figure legend, table legend, main text, or Methods section.

n/a	Confirmed	
☐	☒	The exact sample size (*n*) for each experimental group/condition, given as a discrete number and unit of measurement
☐	☒	A statement on whether measurements were taken from distinct samples or whether the same sample was measured repeatedly
☐	☒	The statistical test(s) used AND whether they are one- or two-sided<br>*Only common tests should be described solely by name; describe more complex techniques in the Methods section.*
☒	☐	A description of all covariates tested
☐	☒	A description of any assumptions or corrections, such as tests of normality and adjustment for multiple comparisons
☐	☒	A full description of the statistical parameters including central tendency (e.g. means) or other basic estimates (e.g. regression coefficient) AND variation (e.g. standard deviation) or associated estimates of uncertainty (e.g. confidence intervals)
☒	☐	For null hypothesis testing, the test statistic (e.g. *F*, *t*, *r*) with confidence intervals, effect sizes, degrees of freedom and *P* value noted<br>*Give P values as exact values whenever suitable.*
☒	☐	For Bayesian analysis, information on the choice of priors and Markov chain Monte Carlo settings
☐	☒	For hierarchical and complex designs, identification of the appropriate level for tests and full reporting of outcomes
☐	☒	Estimates of effect sizes (e.g. Cohen's *d*, Pearson's *r*), indicating how they were calculated

Our web collection on statistics for biologists contains articles on many of the points above.

Software and code

Policy information about availability of computer code

Data collection	No software was used
Data analysis	Metaboanalyst software (Chong et al, 2019); Timetree software (Kumar et a, 2017); RStudio software (v1.1.453) and its packages "Hmisc" (Harrel Jr and Dupont, 2020), "corrplot" (Wei and Smiko, 2017), "ggplot2" (Wicham, 2016), "caper" (Orme et al, 2018); GraphPad Prism software (v8.0.1)

For manuscripts utilizing custom algorithms or software that are central to the research but not yet described in published literature, software must be made available to editors and reviewers. We strongly encourage code deposition in a community repository (e.g. GitHub). See the Nature Research guidelines for submitting code & software for further information.

Data

Policy information about availability of data

All manuscripts must include a data availability statement. This statement should provide the following information, where applicable:

- Accession codes, unique identifiers, or web links for publicly available datasets
- A list of figures that have associated raw data
- A description of any restrictions on data availability

All data generated or analysed during this study are include in this published article (and its supplementary information files)

Field-specific reporting

Please select the one below that is the best fit for your research. If you are not sure, read the appropriate sections before making your selection.

☒ Life sciences ☐ Behavioural & social sciences ☐ Ecological, evolutionary & environmental sciences

For a reference copy of the document with all sections, see nature.com/documents/nr-reporting-summary-flat.pdf

Life sciences study design

All studies must disclose on these points even when the disclosure is negative.

Sample size	Choosing the appropriate sample size for high-throughput approaches involving multivariate data is complicated. A pilot study to define the sample size would be needed but it is not realistic for their economic cost. In practice, ethical and economical restrictions mainly determine the number of samples (i.e., animals) for each group (Vinaixa et al. Metabolites. 2012 Dec; 2(4): 775–795.). For the present study, sample size was elected by ethical and economic restrictions and conditioned to sample availability.
Data exclusions	No data were excluded
Replication	To verify the reproducibility, samples were obtained from diferent specimens (from 4 to 6, depending on sample availability) and individuals (n=6, to be similar to the number of samples obtained from specimens)
Randomization	Prior sample analyses, all samples were randomized.
Blinding	Blinding was not relevant sice samples were randomized prior metabolomic analyses.

Reporting for specific materials, systems and methods

We require information from authors about some types of materials, experimental systems and methods used in many studies. Here, indicate whether each material, system or method listed is relevant to your study. If you are not sure if a list item applies to your research, read the appropriate section before selecting a response.

Materials & experimental systems

n/a	Involved in the study	
☒	☐	Antibodies
☒	☐	Eukaryotic cell lines
☒	☐	Palaeontology and archaeology
☐	☒	Animals and other organisms
☐	☒	Human research participants
☒	☐	Clinical data
☒	☐	Dual use research of concern

Methods

n/a	Involved in the study	
☒	☐	ChIP-seq
☒	☐	Flow cytometry
☒	☐	MRI-based neuroimaging

Animals and other organisms

Policy information about studies involving animals; ARRIVE guidelines recommended for reporting animal research

Laboratory animals	Mammalian species included in the study were male adult specimens with an age representing their 15-30% of their longevity. The recorded values for longevity (in years) were: mouse (Mus musculus, n=4), 3.5; rat (Rattus norvegicus, n=5), 4.5; guinea pig (Cavia porcellus, n=5), 8; rabbit (Oryctolagus cuniculus, n=5), 13; sheep (Ovis aries, n=5), 20; dog (Canis lupus, n=5), 24; pig (Sus scrofa, n=5), 27; cow (Bos taurus, n=4), 30; cat (Felis catus, n=5), 30; horse (Equus caballus, n=5), 46; and human (Homo sapiens, n=6), 120.
Wild animals	Rodents and rabbits were obtained from rodent husbandries and sacrificed by decapitation, whereas plasma from dogs, cats, sheeps, pigs, cows, and horses were obtained from farms. For humans, plasma samples were obtained from healthy adult individuals.
Field-collected samples	Rodents and rabbits were maintained on standard housing conditions.
Ethics oversight	The animal care protocols were approved by the Animal Experimentation Ethics Committee of the University of Lleida.

Note that full information on the approval of the study protocol must also be provided in the manuscript.

Human research participants

Policy information about studies involving human research participants

Population characteristics	Healthy individuals between 30-50 years old
Recruitment	We randomly selected the human samples from a healthy population from 30-50 years old.
Ethics oversight	Human protocols were approved by the Committee for Ethics in Clinical Research of the Hospital Universitari Arnau de Vilanova, in accordance with the Declaration of Helsinki.

Note that full information on the approval of the study protocol must also be provided in the manuscript.
